# Brain Endothelial Cells Release Apical and Basolateral Microparticles in Response to Inflammatory Cytokine Stimulation: Relevance to Neuroinflammatory Stress?

**DOI:** 10.3389/fimmu.2019.01455

**Published:** 2019-06-27

**Authors:** J. Winny Yun, Mansoureh Barzegar, Christen J. Boyer, Alireza Minagar, Pierre-Olivier Couraud, Jonathan Steven Alexander

**Affiliations:** ^1^Department of Molecular and Cellular Physiology, Louisiana State University Health Sciences Center, Shreveport, LA, United States; ^2^Department of Neurology, Louisiana State University Health Sciences Center, Shreveport, LA, United States; ^3^CNRS UMR8104, Institut Cochin, INSERM U1016, Université Paris Descartes, Paris, France

**Keywords:** inflammation, neurovascular, microparticle, extracellular vesicle, brain, endothelial

## Abstract

Microparticles (MP) are regarded both as biomarkers and mediators of many forms of pathology, including neurovascular inflammation. Here, we characterized vectorial release of apical and basolateral MPs (AMPs and BMPs) from control and TNF-α/IFN-γ treated human brain endothelial monolayers, studied molecular composition of AMPs and BMPs and characterized molecular pathways regulating AMP and BMP release. The effects of AMPs and BMPs on blood-brain barrier properties and human brain microvascular smooth muscle tonic contractility *in vitro* were also evaluated. We report that human brain microvascular endothelial cells release MPs both apically and basolaterally with both AMP and BMP release significantly increased following inflammatory cytokine challenge (3.5-fold and 3.9-fold vs. control, respectively). AMPs and BMPs both carry proteins derived from parent cells including those in BBB junctions (Claudin−1, −3, −5, occludin, VE-cadherin). AMPs and BMPs represent distinct populations whose release appears to be regulated by distinctly separate molecular pathways, which depend on signaling from Rho-associated, coiled-coil containing protein kinase (ROCK), calpain as well as cholesterol depletion. AMPs and BMPs modulate functions of neighboring cells including BBB endothelial solute permeability and brain vascular smooth muscle contractility. While control AMPs enhanced brain endothelial barrier, cytokine-induced AMPs impaired BBB. Cytokine-induced but not control BMPs significantly impaired human brain smooth muscle contractility as early as day 1. Taken together these results indicate that AMPs and BMPs may contribute to neurovascular inflammatory disease progression both within the circulation (AMP) and in the brain parenchyma (BMP).

## Introduction

Microparticles (MPs) are 0.1–1.0 μm diameter cell membrane-derived vesicles formed by direct budding from the cell surface (1). In unstimulated cells, the anionic phospholipid phosphatidylserine (PS) is restricted primarily to the inner cytoplasmic leaflet of the plasma membrane. During endothelial cell activation by inflammatory mediators PS is externalized to the outer cell membrane, where it is concentrated and released from cells in MPs. Consequently, the presence of PS on the external surface of MPs (along with appropriate size gating) has been used to detect and quantify MPs using flow cytometric binding of fluorescently tagged Annexin V to MP-associated PS (2). Besides externalized PS, MPs also retain integral membrane proteins, mRNAs, miRNAs and cytosolic proteins derived from their originating “parent” cells. Consequently, MPs are often considered to be “bioactive,” potentially contributing to disease activity and progression as well as serving as a marker of cell activation (3–5).

In the vasculature, many cell types can generate MPs including platelets (6), monocytes (7), and endothelial cells (ECs) (8). EC-derived microparticles (“EMPs”) are constitutively released at low levels under normal physiological conditions, with the rate and magnitude of EMP release increasing during endothelial inflammatory activation or apoptosis (8). Several recent studies analyzing circulating MPs in plasma and serum samples have implicated MPs in the pathology of neurodegenerative, neurovascular and inflammatory diseases including stroke (9), multiple sclerosis (MS) (10) and Alzheimer's disease (AD) (11, 12). Apically released EMPs have widely been reported, both in *in vitro* and *in vivo* studies as well as in clinical studies (1, 10). Such MPs accumulate in the plasma and serum *in vivo*, and in cell culture media *in vitro*. However, so far, there have been no reports on basolaterally released EMPs or “BMPs.”

**Figure 1:**
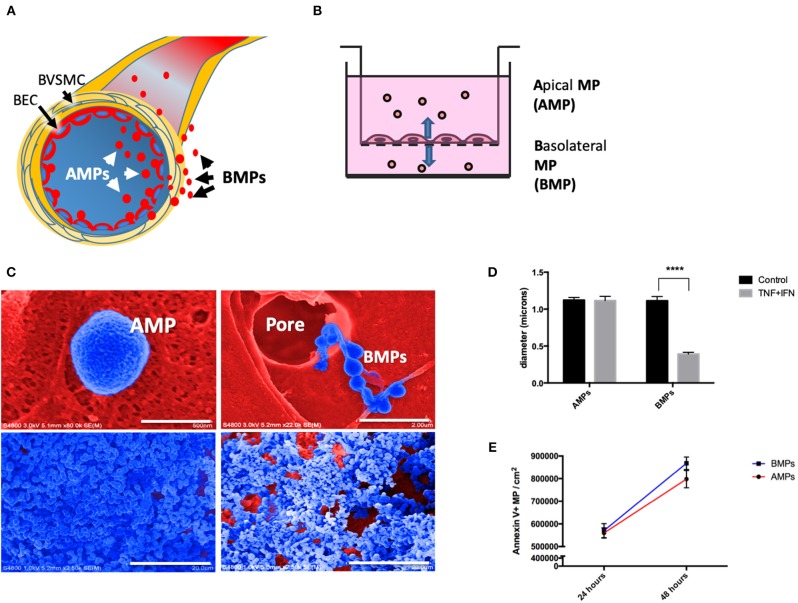
Brain endothelial cells release microparticles both apically and basolaterally. **(A)** Brain endothelial cell (BEC) release MPs apically (AMPs) into the vascular space and basolaterally (BMPs) into the paravascular space. Once released, BMPs can interact with brain vascular smooth muscle cells (BVSMC) to affect the contractility and therefore vasomotion. **(B)** Schematic drawing representing the *in vitro* set up using 3um pore transwell insert for the collection of AMPs, BMPs, and cells. **(C)** Ultrastructural appearance of human brain endothelial MPs by scanning electron microscopy. Top left: Close-up appearance of AMPs showing crenulated surface (blue, filter matrix is red). Top right: shown MPs passing through 8 um pore, demonstrating that endothelial cells transfer microparticles to the basolateral domain. Bottom left: AMPs captured by centrifugation on PVA capture matrix, Bottom right: BMP on capture matrix. **(D)** Diameter of AMPs and BMPs under control and stimulated conditions. **(E)** Quantification of AMPs and BMPs under unstimulated condition at 24 and 48 h. ^****^p < 0.0001.

Here, in addition to studying apically-released microparticles (AMPs), we also for the first time describe the release of basolaterally-directed MPs or “*BMP's”* (Figure 1A). The *in vitro* release of BMPs may indicate that EMPs are also released into perivascular spaces where they may modulate functions of adventitial cells (e.g., smooth muscle).

The objectives of the present study were to (1) to evaluate vectorial (apical and basolateral) release of MPs from human cerebrovascular endothelium, (2) measure amounts of apical and basolateral MPs released following exposure to inflammatory cytokines, (3) identify molecular components of AMPs and BMPs (compared to the parent monolayer), (4) describe molecular pathways regulating AMP vs. BMP release, (5) describe BMP interactions with and regulation of cerebrovascular smooth muscle contractility, and (6) describe AMP effects on endothelial barrier function. These distinct and different populations of EMPs suggests an additional layer of complexity, that have not previously been thought of, which may be important in health and disease.

## Materials and Methods

### Cell Culture

Human cerebral micovascular endothelial cells (hCMEC/D3 cell line) were provided by Dr. P.O Couraud (INSERM, France). hCMEC/D3 (hereafter “D3”) were cultured on rat tail type I collagen (0.1 mg/ml)-coated flasks (Corning, Corning, NY) in “complete” growth medium [EndoGRO^TM^-MV Complete culture media kit (Millipore, Burlington, MA), 1% Penicillin-Streptomycin (Cellgro, Swedesboro, NJ)] at 37°C in 5% CO_2._ D3 cells were used between passages 27 and 35.

### Apical and Basolateral Microparticle Culture

To collect and study endothelial microparticles (EMP), hCMEC/D3 were plated onto 3 um pore 6 well transwell plates (Corning) in 2 ml of “complete' media added to the bottom (basolateral chamber) of each well and 1.5 ml of medium added to the top of each transwell insert (apical) which contained cells (Cells cultured in this manner did not migrate between compartments as verified by crystal violet staining and scanning electron microscopy). In order to study the effects of inflammatory cytokines on EMP release in each compartment, after cells had reached confluency for 48 h, media in both compartments were replaced with media containing 1,000 U/ml of interferon-gamma (IFN-γ, Thermofisher Scientific, Rockford, MA) and/or 20 ng/ml of tumor necrosis factor-alpha (TNF-α, Thermofisher Scientific) or control medium. Media were separately removed from apical and basolateral compartments to isolate MP at 24 and 48 h following treatment. All experiments were normalized to equal surface area of D3 cells used to produce MPs. Following collection of medium for MP isolation, cells were lysed in radioimmunoprecipitation assay buffer (RIPA buffer, ThermoFisher Scientific) for subsequent Western blotting analysis.

### Microparticle Isolation

Following exposure of D3 cells to control medium or medium supplemented with IFN-γ/TNF-α, (described in “apical and basolateral microparticle culture”) culture media were collected by centrifugation. Unattached cells and debris were initially removed by centrifugation at 400xg for 10 min at 4°C and supernatants transferred to fresh microcentrifuge tubes and re-centrifuged at 20,800 g for 1 h at 4°C to pellet MPs. Supernatants were carefully aspirated and MP pellets washed twice by centrifugation using 4°C PBS plus 1 mM phenylmethylsulfonyl fluoride (PMSF) again at 20,800 g for 15 min at 4°C. Supernatants were aspirated, and MP pellets stored at −80°C until analyzed.

### Flow Cytometry Analysis

To evaluate microparticles released by D3 cells using flow cytometry, freshly isolated MPs were resuspended in 100 μl Annexin V Binding Buffer (BD Biosciences, San Jose, CA) (10 mM Hepes, 140 mM NaCl, 2.5 mM CaCl_2_) plus 5 μl of Annexin V-FITC (BD Biosciences) for 1 h at 4°C under light-protected conditions. Nine hundred microliter of 1X “Binding Buffer” was added to each sample. These samples were immediately analyzed by flow cytometry using a BD FacsCaliber (BD Biosciences) instrument.

MP flow cytometric analysis was calibrated using 0.5, 1, and 2 um size Fluorsbrite^TM^ Yellow Green Microspheres (Polysciences, Warrington, PA). Logarithmic scale side scatter plots of sizing beads were used to determine appropriate gating for the MP samples and to select for MPs that were >0.5 um and <1 um in size.

### Western Blotting

To prepare whole cell and MP lysates from D3 cells cultured in six-well transwell plates, media were removed from the top and bottom chambers and MPs isolated as described. Cells were washed with PBS and lysed in 100 μl of Laemmli buffer (Biorad, Hercules, CA) per insert. Cell lysates were then harvested by cell scraping, lysates were sonicated for 15 s, boiled and frozen at −80°C. For MP western blotting, MP pellets were also lysed in Laemmli buffer, sonicated, boiled, and frozen at −80°C. Membranes were probed with rabbit anti-caveolin-1 [1:1000, Cell Signaling Technologies (CST), Danvers, MA], rabbit anti-β-tubulin (1:1000, CST), rabbit anti-claudin-1 (1:1000, CST), rabbit anti-claudin-3 (1:1000, Abcam, Cambridge, MA), rabbit anti-claudin-5 (1:5000, Abcam), rabbit anti-occludin (1:1000, Abcam), and rabbit anti-VE-cadherin (1:1000, Abcam). Biorad ECL reagents were added to membranes, which were then developed on blue x-ray film (Phenix Research Products, Candler, NC). Densitometry was performed using ImageJ software analysis (NIH).

### Scanning Electron Microscopy

Confluent monolayers on 3 μm pore size transwell plates (Corning) were treated with media containing 1,000 U/mL of IFN-γ plus 20 ng/mL of TNF-α or with media control. Forty-Eight hours post treatment media were removed from the apical transwell insert and the basolateral compartment and MPs pelleted in tubes containing cross-linked polyvinyl alcohol platforms. Both cells on inserts and MP pellets on PVA platforms were then fixed in 2% glutaraldehyde overnight. Samples were next dehydrated in graded series of alcohols for 15 min each (50–100%). Following dehydration samples underwent alternative critical point drying using hexamethyldisilazane (HMDS) (13). For scanning electron microscopy (SEM) MP samples were mounted on double-sided adhesive carbon tape and attached to the working SEM stage. Gold sputter coating (4 nm) was applied to the collected MP surfaces using a Cressington 208 HR Metal Sputter Coater (Watford, England). MP surface topographies were recorded using an S-4800 field-emission SEM (HITACHI, Tokyo, Japan).

### Permeability Assay

To evaluate the effects of EMPs on brain endothelial barrier function, an avidin permeation assay was performed after exposing monolayers to AMPs and cytokines. A biotinylated gelatin stock solution was prepared by mixing Ez-link-biotin (ThermoFisher) dissolved in DMSO (5.7 mg/ml) and then mixed with gelatin [dissolved in NaHCO3 buffer (10 mg/ml), pH 8.3]. Biotinylated gelatin stock solution was diluted in NaHCO3 buffer (1:40 dilution) before adding to a 12-well plate and incubated at 4°C overnight. After removing the biotinylated gelatin solution, the wells were washed with 1X PBS two times. D3 cells were plated and allowed to reach confluency. Monolayers treated with TNF-α (20 ng/ml), IFN-γ (1,000 U/ml), TNF-α+IFN-γ (20 ng/ml + 1,000 U/ml), or MPs collected from TNF-α (20 ng/ml), IFN-γ (1,000 U/ml), TNF-α+IFN-γ (20 ng/ml + 1,000 U/ml)- treated D3 cells for 24 h. 1:50 dilution of FITC-avidin (Life Technologies-Molecular Probes; 434411) was added directly to the media and incubated for 3 min at 37°C protected from light. The media was removed and the wells were washed with warm 1X PBS two times. Cells were fixed with 4% paraformaldehyde for 10 min and imaged using Nikon E600FN.

### Collagen Gel Contraction Assay

Collagen gel contraction assays were used to study the effects of BMPs on human brain smooth muscle tonic contractility as previously described (14). Briefly, collagen was solubilized in 0.012M HCL and hBVSMCs (Sciencecell, Carlsbad, CA) were harvested with trypsin-EDTA and re-suspended in DMEM at a volume to give 1 × 10^5^ cells/ml. 5X DMEM was added to acid solubilized collagen to reach a final concentration of 1.25 mg/ml collagen. The gel acidity was immediately neutralized to pH ~7.4 with 1N NaOH. hBVSMCs (50,000 cells/gel) mixed with collagen were added to 24-well plates at 0.5 ml of collagen-cell mix/well) and incubated for 1 h at 37°C in 5% CO_2_ for 1 h to polymerize collagen. One milliliter of DMEM (with or without BMP) were added to each well. Gels were detached from the wall of the wells using a sterile glass pipette and incubated at 37°C in 5% CO_2_ and 95% air over 6 days. All experiments were performed in triplicate. At each time point, photographs of gels were recorded and the area of the gel in each well were analyzed by NIH Image J Imaging analysis program. Data are presented as area in mm^2^.

### Statistical Analysis

Data are expressed as mean ± standard error of the mean (SEM). Differences between groups were analyzed using Student's *t*-test, one- or two-way ANOVA, (with Bonferroni post-test) where appropriate and indicated in the figure legend. A *p*-value < 0.05 was considered statistically significant.

## Results

### Brain Endothelial Cells Release Both Apical and Basolateral Microparticles

To evaluate apical and basolateral release of brain EMPs we separately collected these two MP populations from D3 cells grown on 3 μm transwell inserts. This culture model allows passage of 0.1–1 μm MPs, but restricts cell passage. MPs collected from the “inside” of the insert represent apical EMPs (AMPs) while MPs collected from the media in the lower chamber represent basolateral EMPs (BMPs) (Figure 1B). Scanning electron microcopy reveals AMPs “budding” off of cells on the apical surface (Figure 1C, top left); in contrast BMPs were observed as “beads on a string” passing through transwell membrane pores (Figure 1C, top right). AMP and BMP pellets collected on cross-linked PVA and visualized by SEM show that morphologically, AMPs are rounder, smoother and more uniform in shape, whereas BMPs appear rougher and more irregular (Figure 1C, bottom panels).

SEM images were used to measure the diameters of AMPs and BMPs, which were not significantly different under unstimulated conditions, averaging 1.12 ± 0.21 μm and 1.11 ± 0.31 μm, respectively. Although diameters of cytokine-induced AMPs were not significantly different from controls, diameters of BMPs significantly decreased (from 1.11 ± 0.31 μm to 0.392 ± 0.133 μm) (Figure 1D).

We next quantified numbers of AMPs and BMPs released by untreated cells at 24 and 48 h using flow cytometry. We found that 559,823 ± 22,703 AMPs and 570,207 ± 30,990 BMPs were released (per cm^2^ of EC surface) into the media by 24 h. We also found that both AMPs and BMPs continued to be released and were both significantly increased by 48 h to 798,082 ± 38,236 and 868,236 ± 27,617, respectively (Figure 1E). Therefore, brain endothelial cells constitutively release MPs both apically and basolaterally.

### Inflammatory Cytokine Treatment Increases MP Release From Brain Endothelial Cells Both Apically and Basolaterally

**Figure 2:**
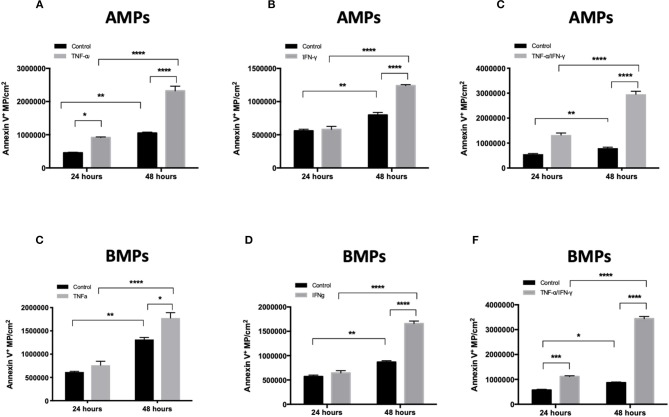
Inflammatory cytokine treatment increases MP release both apically and basolaterally. Quantification of MP released from hCMEC/D3 cells in culture using flow cytometry. AMPs and BMPs were quantified following 48 h TNF-a **(A)**, IFN-g **(B)**, and combined **(C)** treatments. Data are presented as the mean ± SEM and analyzed with two-way ANOVA followed by Bonferroni test. ^*^p < 0.05, ^***^p < 0.001, ^****^p < 0.0001.

To evaluate effects of inflammatory cytokines on the magnitude of brain endothelial EMP release as a model of neurovascular inflammation, D3 cells were treated with control medium or medium supplemented with TNF-α, IFN-γ or a combination of TNF-α plus IFN-γ (T/I). AMPs and BMPs were then quantified by flow cytometry (Figure 2).

EMPs (both AMPs and BMPs) were released by both control and cytokine-treated cells. TNF-α, IFN-γ, and TNF-α/IFN-γ treatments each significantly increased numbers of Annexin V^+^ EMPs released by brain endothelial cells compared to controls (Figures 2A–C), demonstrating that cerebrovascular inflammatory stress increases both apical and basolateral EMP production by brain endothelium. EMPs collected and quantified at 24 and 48 h after cytokine addition show that both AMP and BMP release increases with the duration of cytokine treatment. Further, although the size of cytokine-induced AMPs remained similar to that of controls (1.115 ± 0.05 μm), the size of BMPs was significantly decreased following T/I treatment, from an average of 1.11 ± 0.31 μm to a mean diameter of 0.392 ± 0.133 μm (Figure 1D). Therefore, inflammatory cytokine stimulation increases brain endothelial release of AMPs and BMPs. However, while the size of AMPs does not change, BMP sizes are reduced after stimulation. This could increase the surface area-to-volume ratio of these microparticles that come into contact and interact with possible recipient cells.

### AMPs and BMPs Contain Brain Endothelial Proteins Including Junctional Proteins

**Figure 3:**
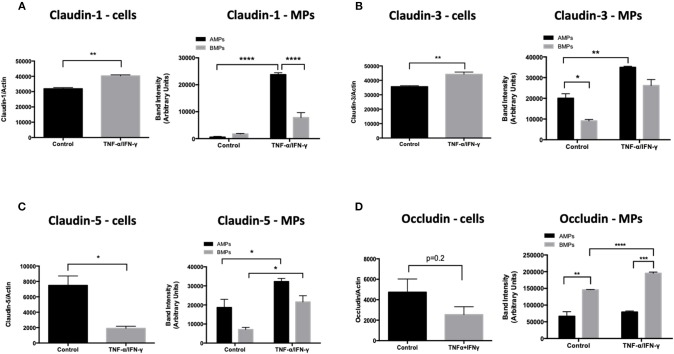
AMPs and BMPs contain tight junction proteins. **(A–D)** Following 24 h of T/I treatment, Claudin-1 **(A)**,−3 **(B)**,−5 **(C)**, Occludin **(D)** expressions were assessed by western blot. Data are presented as the mean ± SEM and analyzed with *t*-test for cells and one-way ANOVA followed by Bonferroni test for MPs. ^*^p < 0.05, ^**^p < 0.01, ^***^p < 0.001, ^****^p < 0.0001.

**Figure 4:**
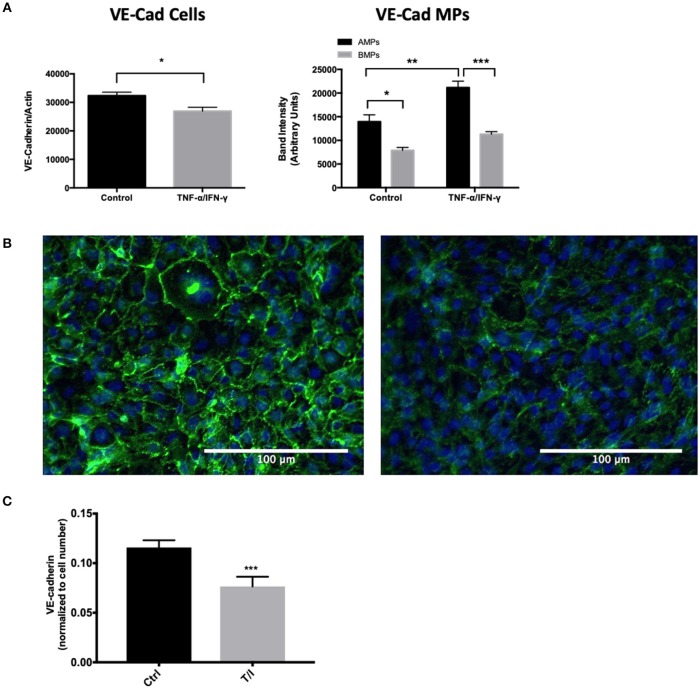
AMPs and BMPs contain adherens junction proteins. **(A)** VE-Cadherin expression assessed by western blot after 24 h of T/I treatment. **(B)** Representative immunofluorescence images visualizing VE-cadherin in the control or 24-h T/I-stimulated hCMEC/D3 [DAPI (4′,6-diamidino-2-phenylindole, dihydrochloride); nuclei=blue, VE-Cadherin=green] and graph quantifying the staining intensity of VE-cadherin in hCMEC/D3. **(C)** Quantification of immunofluorescence images of VE-cadherin in the control or 24-h T/I-stimulated hCMEC/D3. Data are presented as the mean ± SEM and analyzed with *t*-test for cells and one-way ANOVA followed by Bonferroni test for MPs. ^*^p < 0.05, ^**^p < 0.01, ^***^p < 0.001.

Endothelial exposure to inflammatory cytokines like TNF-α and IFN-γ alone and in combination can perturb BBB (15, 16) by reducing the brain endothelial cell content of tight and adherens junctional components (17). We therefore investigated the transmission of junctional proteins involved in BBB establishment into MPs following T/I treatment. Western blotting revealed an increased abundance of Claudins−1,−3, and−5 within both AMPs and BMPs following T/I treatment at 24 h (Figures 3A–C). We also found an increased abundance of occludin in BMPs derived from T/I treated D3 cells (Figure 3D). T/I treatment reduced Claudin-5 content in the “parent” cells, however levels of Claudins−1 and−3 in cells were significantly increased. We also found significantly reduced expression of VE-cadherin, an important adherens junction component of the BBB, by both Western blotting (Figure 4A) and by immunofluorescence (Figure 4C). Interestingly, VE-Cadherin was significantly increased in both AMPs and BMPs, with net higher expression in AMPs compared to BMPs (Figure 4B) consistent with transfer of VE-Cadherin from parent cells to MPs.

### AMPs From Brain Endothelial Cells Alter Brain Endothelial Solute Permeability

**Figure 5:**
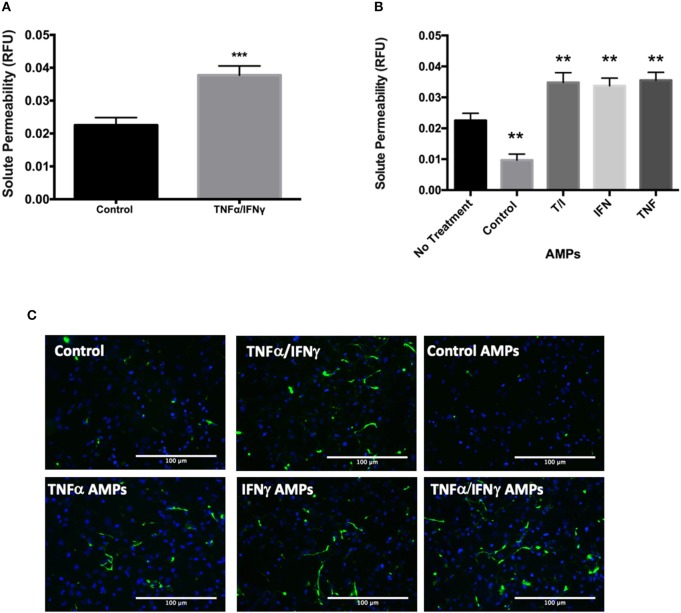
AMPs from brain endothelial cells alter BBB barrier function. **(A)** Analysis of D3 monolayer permeability for FITC-avidin following TNF+IFN stimulation as relative fluorescence unit (RFU) normalized to DAPI. Data are presented as the mean ± SEM; *n* = 3, and analyzed with *t*-test. ^***^p < 0.001 **(B)** Analysis of D3 monolayer permeability for FITC-avidin following incubation with AMPs isolated from D3 monolayers with or without cytokine stimulation. Data are presented as the mean ± SEM; *n* = 3, and analyzed with one-way ANOVA followed by Bonferroni test. ^**^p < 0.01 **(C)** Representative immunofluorescence images visualizing FITC-avidin binding to gelatin underlying the D3 monolayer indicating extent of EC barrier dysfunction in the control or D3 cells stimulated with T/I or D3 AMPs.

The transfer of junctional proteins from D3 cells into MPs following cytokine treatment was also associated with increased solute permeability of T/I treated D3 monolayers (Figure 5A). Interestingly, when endothelial monolayers were incubated with AMPs isolated from *unstimulated* D3 cells, solute permeability was significantly *decreased*, indicating enhanced barrier function (Figure 5B). Conversely the addition of AMPs isolated from D3 cells treated with TNF, IFN or combined cytokine treatment each significantly increased permeability (Figure 5B), indicating that AMPs can exert protective or harmful effects on endothelial barrier function depending on the conditions under which they were released i.e., basal or cytokine-activated.

### BMPs From Cytokine- Inflamed Brain Endothelial Cells Decrease Brain Vascular Smooth Muscle Contractility

**Figure 6:**
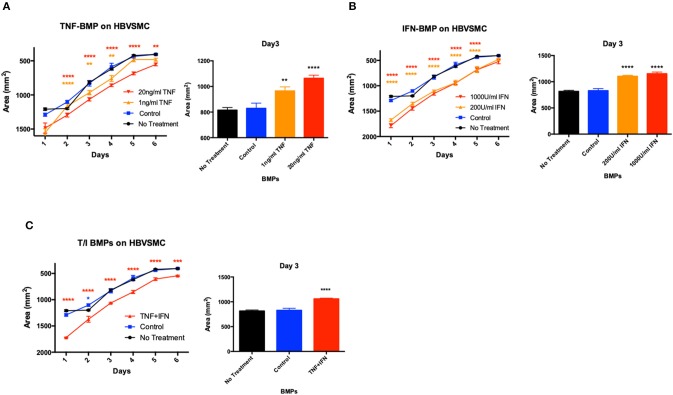
BMPs from inflamed brain endothelial cells decrease brain vascular smooth muscle contractility. HBVSMC area was analyzed over 6 days after treatment with BMPs collected from TNF-a **(A)**, IFN-g **(B)**, and T/I **(C)** treated D3 cells. Data are presented as the mean ± SEM and analyzed one-way ANOVA followed by Bonferroni test. ^*^p < 0.05, ^**^p < 0.01, ^***^p < 0.001, ^****^p < 0.0001.

Because smooth muscle cells are important adventitial components of the BBB, BMPs released into the perivascular space could modulate vascular contractility and blood flow. We investigated whether and to what extent BMPs released by brain endothelial cells modulate contractility of brain vascular smooth muscle cells. BMPs released from D3 cells treated with TNF-α, IFN-γ or T/I were collected and added to brain vascular smooth muscle cells in collagen gels, a model of vascular tonic contractility (14). While BMPs isolated from unstimulated D3 cells did not significantly alter the contractile capacity of brain vascular smooth muscle, BMPs from TNF-treated D3 cells significantly reduced tonic contractility (Figure 6A). Similarly, BMPs from D3 cells treated IFN-γ or combined cytokine treatments (1 ng/ml TNF and 200 U/ml IFN-γ) also significantly reduced brain smooth muscle contraction (Figures 6B,C).

### Regulation of AMP and BMP Release by Distinct Molecular Pathways

**Figure 7:**
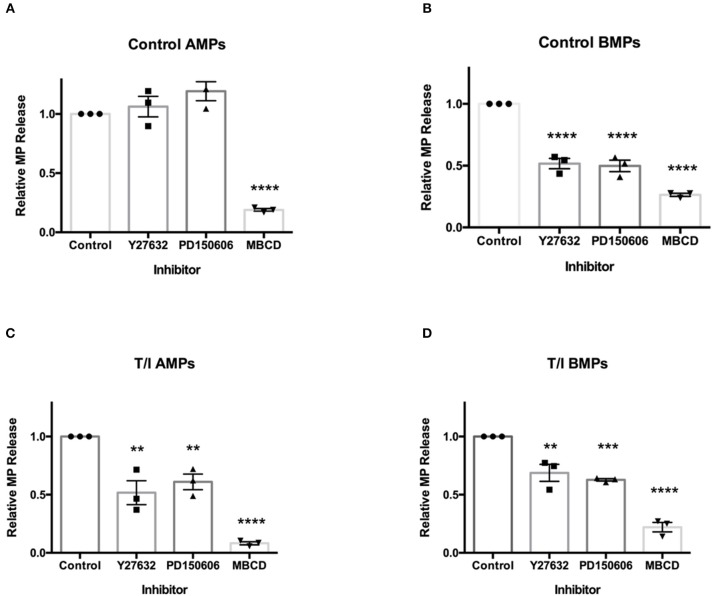
Secretion of AMPs and BMPs are regulated by distinct molecular pathways. Effects of various inhibitors on D3 AMP and BMP formation. Cells were pre-treated with ROCK inhibitor Y27632 (30 μM, 2 h), calpain inhibitor PD150606 (2 μM, 1) or lipid-rich microdomain disrupting agent MBCD (10 mM, 45 min) prior to 24-h T/I treatment. AMPs **(A,C)** and BMPs **(B,D)** were collected and quantified. Data are presented as the mean ± SEM and analyzed one-way ANOVA followed by Bonferroni test. ^**^p < 0.01, ^***^p < 0.001, ^****^p < 0.0001.

To evaluate cell signaling programs controlling AMP and BMP generation, several molecular mechanisms known to influence the release of MPs were investigated. Several studies have shown that MP secretion is regulated by Rho kinase (ROCK) (18–21), calpain (22–24), requires caveolar components (18) including lipid rafts (18, 25, 26). To determine whether and which of these molecular pathways were involved in the release of AMPs and BMPs from unstimulated and cytokine-stimulate D3 cells, transwell cultured D3 cells were pre-treated with 30 uM Y27632 (an inhibitor of ROCK I and II), 2 uM PD150606 (an inhibitor of calpain), or 10 mM methyl-β-cyclodextrin (MBCD; a cholesterol depleting agent which disrupts lipid rafts), in control media or media containing TNF-α/IFN-γ for 24 h. AMPs and BMPs were harvested and analyzed by flow cytometry (Figure 7).

Under control conditions, AMP release by D3 cells was not affected by Y27632 or PD150606 (Figure 7A). Therefore, basal release of endothelial AMPs appears to only require the membrane cholesterol (an important component of MPs), without which AMPs are not formed. Conversely, under control conditions, the release of BMPs by endothelial cells was significantly reduced by Y27632 and PD150606 as well as by MBCD (Figure 7B). Because BMP release is both ROCK and calpain-dependent under unstimulated conditions, BMP release appears to be mechanistically distinct from that for AMPs.

In the presence of TNF-α/ IFN-γ, AMPs, and BMPs released by D3 cells were significantly reduced by Y27632, PD150606, and MBCD (Figures 7C,D). This indicates that both AMP and BMP release from cytokine-treated cells requires ROCK and calpain in addition to cholesterol availability. These findings show that under unstimulated conditions, the mechanisms controlling AMP release differ from those controlling BMP release. AMP formation from cytokine-treated endothelial cells also differs mechanistically from AMP release by unstimulated endothelial cells.

## Discussion

Research in the field of MP biology has gained tremendous attention over the past decade with MPs being found to be significantly increased in several diseases including MS (10), AD (27), stroke (28), coronary artery disease (29), and type 2 diabetes (4) to name only a few. Consequently, MPs have become useful biomarkers of many diseases, which not only have diagnostic and prognostic value, but also provide mechanistic insights (1, 30). Further, because MPs carry many elements of the “parent” cell (including mRNAs, miRNAs and cytosolic and integral membrane proteins), MPs are also “*bioactive*,” and may transfer materials and/or induce remote signaling in recipient cells to actively influence disease progression (3–5). Despite the exponential increase in MP research, MP studies have only described apically released MPs, that disperse within the vascular space into plasma or serum samples *in vivo* (or in cell culture media *in vitro*).

Here, we describe vectorial release of EMPs by cerebrovascular endothelial cells which are released apically (the “*vascular”* space) as well as basolaterally—into a compartment which would correspond to the “parenchymal” space. Based on their abundance, protein cargo, the molecular programming controlling their formation and their influence on other cell types, AMPs and BMPs appear to represent important, distinctive and biologically active EMP populations. This *in vitro* study considers potential cell behaviors and interactions which may be related to physiology/pathophysiology.

We chose to study the population known as “microparticles” based on the retention of soluble endothelial biomarkers following serial centrifugation at 20,800 g for 1 h, a common protocol used for MP isolation, rather than high speed centrifugation associated with much smaller extracellular vesicles, exosome, isolation (~100,000g). We previously reported that in cell-free plasma samples, greater than 85% of the signal associated with 5 different endothelial biomarkers could be isolated using the same centrifugation protocol which we used in this study (31). Therefore, although we do recognize that exosomes represent a significant and important population, here we have limited our studies to particles which are most consistent with MPs.

Both apically and basolaterally generated MPs were increased in response to cytokine treatments. When normalized to the surface area of D3 cells, on average 3.1 ± 0.12 AMPs and 3.2 ± 0.17 BMPs are released per cell under control conditions, with inflammatory cytokine stimulation increasing this value to 4.4 ± 0.21 and 4.9 ± 0.92, respectively. In terms of their sizes, under basal conditions AMPs and BMPs were similar in diameter (1.12 ± 0.21 μm and 1.11 ± 0.31 μm, respectively), however exposure to inflammatory cytokines significantly reduced diameters of BMPs (to 0.39 ± 0.13 μm), while the diameter of AMPs was unchanged. This indicates that in addition to significantly increasing the numbers of BMPs released following cytokine exposure, the size of BMPs decreases. This reduction in size increases BMP surface area/volume ratio and might enhance binding of BMPs to “recipient” cells in the adventitia.

BMPs, once released “beneath” endothelial monolayers may act as potent modifiers of perivascular cells e.g., vascular smooth muscle. We found that while control BMPs did not affect smooth muscle contractility, BMPs derived from cytokine-treated D3 cells impaired smooth muscle tonic contractility within 24 h. Based on the *in vitro* and *in vivo* (TNF-α-t^1/2^ = 18.2 min, IFN-γ- t^1/2^ = 3 min) lability of cytokines (32, 33), our data appear most consistent with cytokine-induced MPs rather than cytokines, as the cause of these responses.

Since vascular smooth muscle tension in the cerebral vascular wall *in vivo* changes the diameter of cerebral blood vessels *in vivo*, reduced brain smooth muscle contractility in this model might impair normal cerebral vasomotion/autoregulation. Since cerebral autoregulation stabilizes brain blood flow against variations in perfusion pressure, BMPs released by inflamed cerebral endothelium might depress brain smooth muscle contraction *in vivo*, with detrimental effects on neurovascular perfusion. Indeed, several neuroinflammatory diseases, including AD, exhibit impaired cerebral autoregulation (34, 35); cerebral autoregulation is also impaired in mouse models of AD, and in patients with sporadic AD (35).

We observed that AMPs and BMPs also retain tight and adherens junctional components derived from the parent endothelial cells which include claudins-1,-3,-5, occludin and VE-cadherin (Figures 3, 4). Although claudin-1 abundance was similar in AMPs and BMPs under unstimulated conditions, significantly more claudin-1 was detected in AMPs following T/I stimulation, consistent with claudin-1 being exported apically (in AMPs) when endothelial cells are cytokine stimulated. Interestingly, the pattern of junctional protein “export” differs significantly between AMPs and BMPs with VE-cadherin, claudins-1,-3 and−5 being significantly increased in AMPs following cytokine stimulation compared to control AMPs. By comparison, cytokine treatment significantly increased VE-cadherin, claudin-5 and occludin abundance in BMPs (compared to control BMPs), demonstrating distinctive junctional protein segregation between AMPs and BMPs.

The BBB barrier function heavily depends on the homophilic binding between Claudin-5 and VE-Cadherin, which are key components of tight and adherens junctions (36, 37). Both Claudin-5 and VE-Cadherin levels were significantly decreased in D3 cells following inflammatory cytokine treatment, consistent with the transfer of these important BBB junctional proteins from D3 cells into MPs where endothelial solute permeability to avidin (60 kD) was significantly increased at 24 h (Figure 5).

Strikingly, AMPs isolated from unstimulated D3 cells tightened the D3 endothelial barrier, whereas AMPs from cytokine treated D3 cells significantly diminished barrier. Therefore, the conditions under which AMPs and BMPs are formed may determine their relative benefit or pathological properties on the BBB and vascular smooth muscle function.

**Figure 8:**
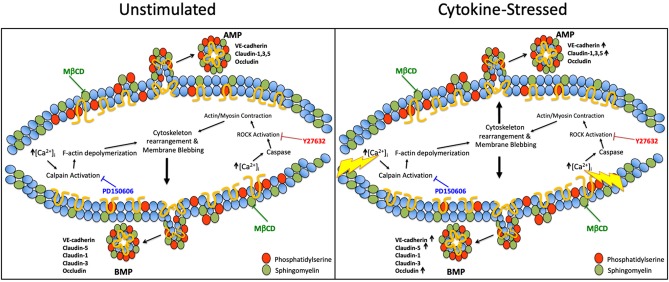
Pathways governing apical and basolateral MP release under unstimulated vs. cytokine-stimulated conditions. Possible pathways governing AMP and BMP release under basal and cytokine-stressed conditions. Under unstimulated conditions, ROCK and calpain are only involved in BMP release however upon cytokine stimulation ROCK and calpain are required for both AMP and BMP generation. Cholesterol/lipid rafts are required in AMPs and BMPs under both basal and cytokine-stimulated conditions.

We also investigated the pathways involved in MP generation from cerebral endothelial cells. Several prior studies have shown that MP secretion from activated cells can be regulated by Rho kinase (ROCK) (18–21) and calpain (22–24) and requires cholesterol to generate MP lipid rafts (18, 25, 26). We also confirmed that the release of BMPs, like that of AMPs, shows a strict requirement for cholesterol availability. However, and additionally, we found that apical and basolateral MP release are governed by different molecular pathways. We also investigated the participation of ROCK and calpain, the two most commonly studied pathways in MP generation in activated endothelial cells. We found that AMP generation, which is independent of ROCK and calpain under unstimulated conditions, became ROCK- and calpain-dependent after inflammatory cytokine challenge. Conversely, BMP release was found to basally require ROCK and calpain, and remained dependent upon ROCK and calpain activity even after cytokine challenge. Therefore, while AMPs and BMPs are both constitutively released, ROCK and calpain are only involved in the continuous release of BMPs, and are only employed for active AMP formation during inflammation (Figure 8). Future studies, which more extensively identify and evaluate mechanisms underlying AMP and BMP release, might permit therapeutic manipulation of AMP and BMP generation and help to explain their contributions in the setting of different diseases.

## Conclusions

Brain endothelial cells release MPs apically and basolaterally under unstimulated conditions and increase the release of these MPs in response to inflammatory cytokine stimulation. BBB junctional proteins are more abundantly “exported” into both of these MP populations following cytokine stimulation. The vectorial release of MPs are also distinctively regulated, which opens up possibilities to specifically influence one subpopulation and not the other.

Because endothelial cells release distinctive populations of MPs in response to inflammatory stimuli with remarkable effects on vascular barrier function and vasomotion, both types of MPs studied here may represent important mediators in health and in disease. An improved understanding of how microparticles are formed and interact with other cells, particularly in the neurovasculature, may provide novel diagnostic, prognostic and therapeutic applications in inflammatory diseases.

## Data Availability

All datasets generated for this study are included in the manuscript and/or the supplementary files.

## Author Contributions

JY: conceived studies, executed experiments, and wrote the study. CB: conceived studies, executed experiments, and helped to write the study. AM: conceived studies, executed experiments, and wrote the study. P-OC: assisted with experimental concepts and design. MB: executed experiments and wrote the study. JA: conceived studies, executed experiments, and wrote the study.

### Conflict of Interest Statement

The authors declare that the research was conducted in the absence of any commercial or financial relationships that could be construed as a potential conflict of interest.
